# Habenular Involvement in Response to Subcallosal Cingulate Deep Brain Stimulation for Depression

**DOI:** 10.3389/fpsyt.2022.810777

**Published:** 2022-02-04

**Authors:** Gavin J. B. Elias, Jürgen Germann, Aaron Loh, Alexandre Boutet, Aditya Pancholi, Michelle E. Beyn, Venkat Bhat, D. Blake Woodside, Peter Giacobbe, Sidney H. Kennedy, Andres M. Lozano

**Affiliations:** ^1^Division of Neurosurgery, Department of Surgery, University Health Network and University of Toronto, Toronto, ON, Canada; ^2^Joint Department of Medical Imaging, University of Toronto, Toronto, ON, Canada; ^3^Centre for Mental Health and Krembil Research Centre, University Health Network, Toronto, ON, Canada; ^4^Centre for Mental Health, University Health Network, Toronto, ON, Canada; ^5^Department of Psychiatry, Sunnybrook Health Sciences Centre, Sunnybrook Research Institute, Toronto, ON, Canada; ^6^Krembil Research Institute, University of Toronto, Toronto, ON, Canada

**Keywords:** habenula, depression, deep brain stimulation, neuroimaging, neuromodulation, treatment biomarker

## Abstract

The habenula (Hb) is a small, evolutionarily conserved epithalamic structure implicated in functions such as reward and mood regulation. Prior imaging work suggests that Hb's structural and functional properties may relate to treatment response in depression and other mood disorders. We used multimodal MRI techniques to investigate the potential involvement of Hb in response to subcallosal cingulate area deep brain stimulation (SCC-DBS) for treatment-resistant mood disorders. Using an automated segmentation technique, we compared Hb volume at baseline and at a subsequent post-operative timepoint (4.4 ± 3.0 years after surgery) in a cohort of 32 patients who received SCC-DBS. Clinical response to treatment (≥50% decrease in HAMD-17 from baseline to 12 months post-operation) was significantly associated with longitudinal Hb volume change: responders tended to have increased Hb volume over time, while non-responders showed decreased Hb volume (*t* = 2.4, *p* = 0.021). We additionally used functional MRI (fMRI) in a subcohort of SCC-DBS patients (*n* = 12) to investigate immediate within-patient changes in Hb functional connectivity associated with SCC-DBS stimulation. Active DBS was significantly associated with increased Hb connectivity to several prefrontal and corticolimbic regions (TFCE-adjusted *p*_Bonferroni_ < 0.0001), many of which have been previously implicated in the neurocircuitry of depression. Taken together, our results suggest that Hb may play an important role in the antidepressant effect of SCC-DBS.

## Introduction

The habenula (Hb) is a small, bilateral, and highly evolutionarily preserved structure situated in the epithalamus (1, 2). Known to play a key role in the regulation of brainstem monoaminergic systems, Hb is broadly implicated in reward processing, social interaction, behavioral adaptation, circadian rhythm, and sensory integration (2–11). It has also been linked to several neuropsychiatric disorders, particularly depression and other mood disorders (3, 7, 9, 12–19). Animal studies have shown that both Hb activity and metrics of reward processing and motivational behavior are altered in models of depression (2, 5, 20, 21). Volumetric studies in humans have likewise detected altered habenula volume in individuals with depression, bipolar disorder, schizophrenia, and autism spectrum disorder (12, 19, 22, 23).

There is also evidence to suggest that Hb plays a role in subserving treatment response in mood disorders. For instance, Hb volume changes have been observed in bipolar disorder (BD) and major depressive disorder (MDD) patients who received pharmacotherapy but not in medication-naïve patients (23). Other studies report response-related changes in Hb functional connectivity in MDD, patients following treatment with electroconvulsive therapy (24) or ketamine (24, 25). Baseline structural and functional Hb connectivity patterns have moreover been shown to predict response (75% sensitivity; 72% specificity) to inpatient treatment in a large MDD cohort (26).

Deep brain stimulation (DBS) is a neuromodulatory technique that employs surgically implanted electrodes to deliver carefully titrated electrical pulses to a specific brain region to modulate brain activity (27, 28). DBS targeting the subcallosal cingulate area (SCC-DBS) is a promising treatment for a variety of psychiatric disorders, including MDD, BD, and anorexia nervosa (AN) (29–39). Evidence from positron emission tomography studies indicates that SCC-DBS not only affects the focal target region but alters activity across distributed circuits in the brain (29, 30, 36). In this study, we employed multimodal MRI techniques to investigate Hb involvement in clinical response to SCC-DBS. Given the putative involvement of Hb in response to antidepressant pharmacotherapy and ECT, we looked at baseline and longitudinal Hb volume in a SCC-DBS cohort, exploring how these variables might relate to clinical outcome. Additionally, in a subcohort of SCC-DBS patients with post-operative functional imaging, we explored how Hb functional connectivity is acutely modified by stimulation.

## Methods

### Design and Patients

This study involved analysis of both retrospectively and prospectively acquired imaging data in psychiatric patients—diagnosed with either major depressive disorder (MDD), bipolar disorder (BD), or anorexia nervosa (AN)—who underwent SCC-DBS therapy for management of depressive symptoms. The eligibility criteria, electrode implantation methods, and post-operative device programming procedure have been previously described (30, 31, 35, 36). All patients received high frequency (130 Hz) stimulation with conventional pulse width settings (60–90 μs). The amplitude/voltage of stimulation and configuration of active electrode contacts were individualized for each patient.

The retrospective component of this study, conducted following institutional research ethics board approval (University Health Network ID: #15-9777), involved review of clinical charts and available structural MR imaging. In keeping with prior retrospective DBS imaging work conducted at our institution (40), we included all patients for whom sufficient clinical data [baseline pre-operative and 12-month follow-up scores on the 17-item Hamilton rating scale for depression (HAMD-17)] and structural MR imaging (pre-operative high-quality scans with complete brain coverage) were available, as long as they had not previously undergone other neurosurgical interventions or received confounding pre- or post-DBS neurological diagnoses. Immediate and—where available—later post-operative structural MR images meeting the aforementioned standards were also collected. This permitted longitudinal analysis of post-DBS brain volume changes in patients for whom ≥2 post-operative scans were available.

The prospective component of this study was conducted with institutional REB approval (University Health Network ID: #14-8255) as part of a publicly registered clinical trial (ClinicalTrials.gov ID: NCT03153670). Here, SCC-DBS patients who were implanted with specific hardware and who were actively using their devices were recruited for functional MRI (fMRI) scanning.

HAMD-17 scores were employed as an index of depression symptom severity. For each patient, percentage improvement from baseline at the 12-month timepoint was computed. In accordance with prior studies, patients with ≥ 50% HAMD-17 reduction were categorized as “responders” (29, 30, 35, 41).

### MRI Acquisition

**Figure 1:**
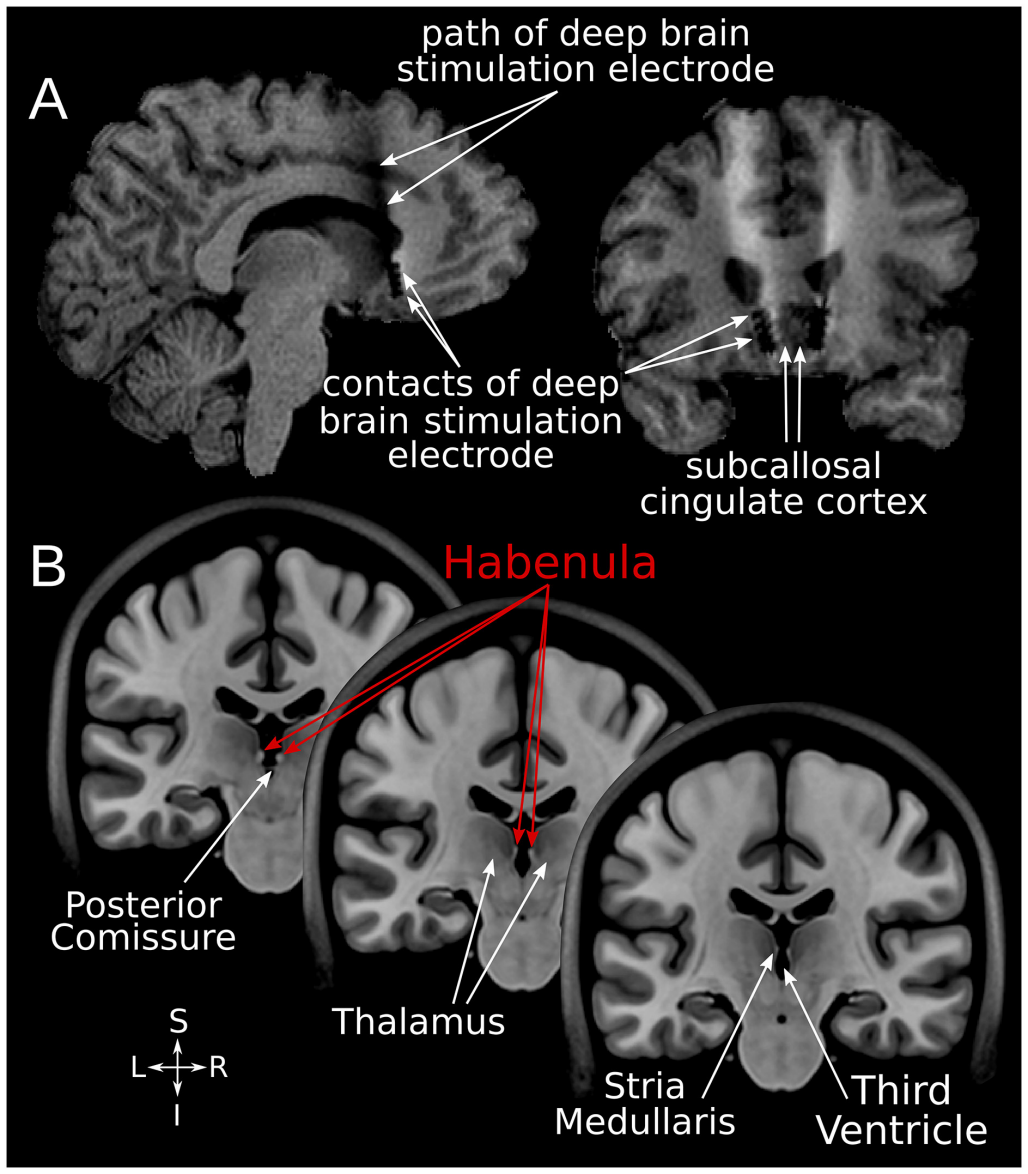
SCC-DBS and the habenula. **(A)** Sagittal and coronal structural T1-weighted MRI slices from an exemplar patient showing the position of the implanted bilateral electrodes. These are placed in the white matter bordering the subcallosal cingulate cortex. **(B)** Coronal slices of the asymmetrical MNI152 template (42) illustrating the location of the habenula and surrounding structures. The habenula appears bright (hyperintense) on T1-weighted MRI. *DBS*, deep brain stimulation; *MNI*, Montreal Neurological Institute; *SCC*, subcallosal cingulate area.

T1-weighted structural MR imaging (Supplementary Table 1) was obtained in all SCC-DBS patients prior to and immediately following DBS implantation to guide surgical planning and confirm electrode placement, respectively (Figure 1A). Additional post-operative structural images were obtained at various later timepoints for clinical purposes or as part of prospective fMRI scanning (see below).

Building on extensive institutional safety testing (43, 44) and prior fMRI scanning in a large cohort of DBS patients (45, 46), we also prospectively obtained 3 Tesla fMRI scans in a subset of SCC-DBS patients following electrode implantation (1.5–13.5 years post-implantation). Only patients who were fully implanted with specific Medtronic DBS hardware (3387 quadripolar leads, 37601 Activa PC, or 37612 Activa RC implantable pulse generators, and 37086 or 7482 extension wires) were considered to be eligible for scanning. The full fMRI paradigm employed here has been described previously (47). Briefly, resting state fMRI (rsfMRI) sequences (Supplementary Table 2) were acquired for each patient while their DBS device was turned on at clinically defined “optimal” settings (DBS-ON) and while it was turned off (DBS-OFF). These sequences, along with a structural scan to facilitate image registration, were acquired in a single MRI session. After changing an individual's DBS settings, we observed a 5-min washout period before beginning the rsfMRI scan.

### Habenular Segmentation and Volumetric Analysis

Using the minc-bpipe preprocessing pipeline (https://github.com/CoBrALab/minc-bpipe-library), all structural MR images were iteratively corrected for non-uniformity, skull-stripped, and rigidly aligned to Montreal Neurological Institute space (MNI 152 ICBM 2009b NLIN asymmetric) without resampling. Subsequently, the bilateral Hb (Figure 1B) was segmented on every image using the automated Multiple Automatically Generated Templates (MAGeT) brain segmentation algorithm (https://github.com/CoBrALab/MAGeTbrain) (12, 48). The hippocampus and its subfields were also segmented bilaterally using MAGeT in order to assess the specificity of any volumetric findings. MAGeT segments structures of interest on individual input images in a contrast-driven fashion *via* registration-based label propagation. It has been shown to be robust for various anatomical structures and subject populations in prior work (12, 19, 48, 49). Specifically, five manually segmented high-resolution atlases are propagated using 21 template images selected from the input dataset, yielding a large number (5 × 21 = 105) of candidate segmentations; these are then fused using a majority vote approach to generate final individualized segmentations. The use of the template library helps to reduce atlas bias and also diminishes registration errors by averaging (49). For quality assurance, each Hb segmentation label was overlaid on the corresponding structural image with DISPLAY (https://www.mcgill.ca/bic/software/minc/minctoolkit) and visually inspected by two raters (JG and GJBE).

### FMRI Functional Connectivity Analysis

Preprocessing of rsfMRI data was conducted with the BRANT toolbox (http://brant.brainnetome.org/) (50). After removing the first 10 volumes, each fMRI sequence was (i) corrected for head motion using volume realignment; (ii) nonlinearly normalized to MNI152 space *via* coregistration to the corresponding structural scan; (iii) resampled to 3 × 3 × 3 mm^3^; (iv) denoised for nuisance variables using a multiple regression model (51); (v) filtered with a temporal bandpass filter (0.01–0.08 Hz); (vi) smoothed with a 6 mm full width at half maximum gaussian kernel. In order to account for the artifact created by the DBS leads and extension wire (45), affected voxels in each individual fMRI image were manually segmented with DISPLAY (https://github.com/BIC-MNI/minc-tools). A group summation map incorporating these individual artifact masks was then created in MNI space; any voxels overlapping this summation map were excluded from further analysis. Finally, the brain-wide functional connectivity of the Hb was examined in each preprocessed rsfMRI image. To do so, we computed Pearson correlations between the average blood oxygen level-dependent (BOLD) time course within a bilateral Hb seed (Supplementary Figure 2) and the BOLD time course of all other voxels in the brain. The resulting connectivity r-maps were then Fisher transformed to z-maps, in which each voxel's value reflected the strength of connectedness between that voxel and the Hb.

### Statistics

The relationship between Hb volume (as derived from MAGeT segmentations) and clinical response status (responder vs. non-responder) was investigated both at pre-operative baseline and longitudinally following DBS implantation. Baseline Hb volume was compared between responders and non-responders using a linear model that controlled for each patient's whole-brain volume (Hb volume ~ response status + whole-brain volume). The interaction between patient-specific Hb volume change over time and response status was investigated *via* a linear mixed-effect model with patient as random intercept [Hb volume ~ time × response status + (1 | patient)]. For the fMRI analysis, changes in Hb functional connectivity between each patient's DBS-ON and DBS-OFF states were examined using voxel-wise paired-tests. Threshold-free cluster enhancement (TFCE) (52) and Bonferroni correction (*p*_Bonferroni_ < 0.0001) were applied to the resulting *t*-map to correct for multiple corrections across the brain. All statistical analyses were performed using R [version 3.6.1; https://www.r-project.org, including the lme4 (version 1.1-21) and lmerTest (version 3.1.1) packages] and RMINC (https://github.com/Mouse-Imaging-Centre/RMINC).

## Results

Overall, 86 SCC-DBS patients, 54 (62.8%) of whom were responders, were included for retrospective analysis of baseline pre-operative Hb volume (Table 1). From this number, 32 patients with serial post-operative structural MR images (80 images overall), 22 (68.8%) of whom were responders, were included for retrospective volumetric analysis (mean duration between baseline and latest post-operative images = 4.4 ± 3.0 years). Twelve of these patients, nine (75.0%) of whom were responders, were also prospectively scanned with 3T fMRI in both DBS-ON and DBS-OFF conditions (mean duration between surgery and fMRI acquisition = 5.6 ± 3.2 years). A study flowchart is provided in the Supplementary Figure 1.

**Table 1:** Demographics, baseline clinical characteristics, and clinical outcome.

**Cohort**	**Age at surgery,** **mean (SD),** **years**	**Sex**	**Baseline HAMD-17** **score, mean (SD)**	**Disease duration** **at surgery,** **mean (SD),** **years**	**Number of** **patients by** **diagnosis (%)**	**Number of** **responders (%)**	**HAMD-17 percentage** **reduction from** **baseline, mean (SD)**
Baseline volume analysis (*n* = 86)	43.8 (10.4)	61 f, 25 m	24.5 (4.9)	22.2 (9.5)	*MDD*: 65 (75.6)	54 (62.8)	53.6 (27.1)
					*BD*: 4 (4.7)		
					*AN*: 17 (19.8)		
Longitudinal volume analysis (*n* = 32)	40.2 (10.7)	26 f, 6 m	23.3 (5.9)	19.7 (7.2)	*MDD*: 16 (50.0)	22 (68.8)	54.4 (30.4)
					*BD*: 2 (6.3)		
					*AN*: 14 (43.8)		
fMRI analysis (*n* = 12)	34.6 (10.1)	11 f, 1 m	23.4 (5.4)	15.7 (7.0)	*MDD*: 3 (25.0)	9 (75.0)	61.1 (26.3)
					*BD*: 1 (8.3)		
					*AN*: 8 (66.7)		

*Responder status and HAMD-17 reduction reported as of 12 months post-operative follow-up. AN, anorexia nervosa; BD, bipolar disorder; HAMD-17, 17-item Hamilton rating scale for depression; MDD, major depressive disorder*.

**Figure 2:**
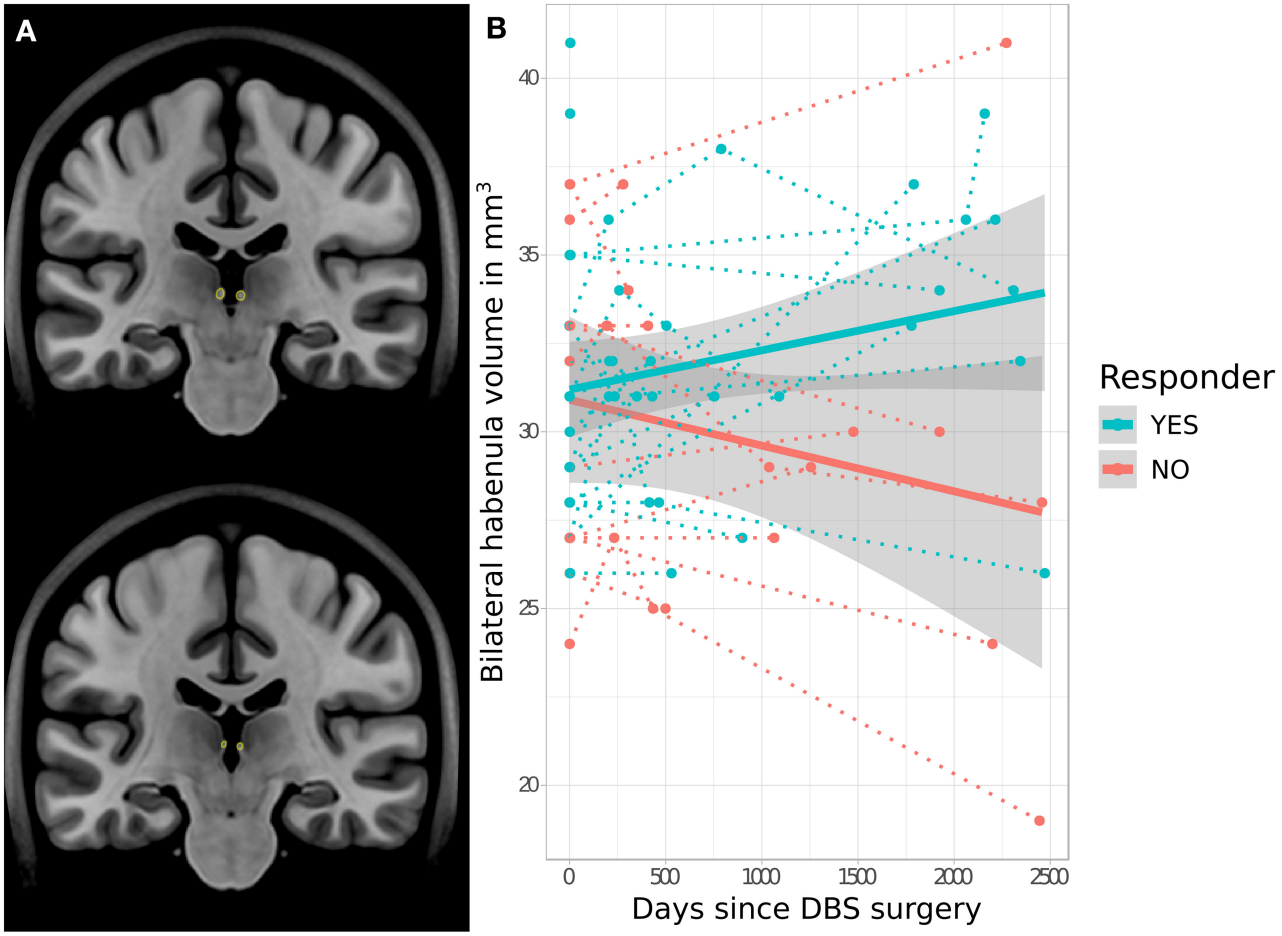
Response-related trajectories of change in habenular volume over time following SCC-DBS. **(A)** Outline of the habenula—as per automated MAGeT segmentation—shown on two different coronal slices of the MNI152 template brain for illustrative purposes. **(B)** Changes in bilateral habenula volume over time following SCC-DBS surgery are shown for each patient (follow-up is cut off at 2,500 days post-surgery for visualization purposes, although some post-operative scans were acquired at later timepoints). The dotted lines indicate the change in habenular volume over time in individual patients, while the thick solid lines indicate the estimated change in habenular volume over time for each cohort overall. The gray shaded zones surrounding the solid lines denote the 95% confidence interval. A significant difference in trajectory of change was found in responders (≥50% HAMD improvement) compared to non-responders: habenula volume decreased over time in non-responders but increased in responders (*t* = 2.4, *p* < 0.021 for interaction of time and response status). *DBS*: deep brain stimulation; *HAMD-17*: 17-item Hamilton rating scale for depression; *MNI*, Montreal Neurological Institute; *SCC*, subcallosal cingulate area.

No significant difference in baseline pre-operative Hb volume was apparent between eventual responders (mean = 30.5 ± 3.4 mm^3^) and non-responders (mean = 30.9 ± 4.9 mm^3^) (*t* = −0.8, *p* = 0.940). However, analysis of longitudinal Hb volume change revealed a significant interaction effect between volume change and response status, with bilateral Hb volume increasing following SCC-DBS surgery in responders but decreasing in non-responders (*t* = 2.4, *p* = 0.021; Figure 2). There was no significant difference between longitudinal cohort responders and non-responders in terms of age (responders: mean = 40.0 ± 10.3; non-responders: mean = 40.7 ± 12.1) or proportion of females (responders: 81.8%; non-responders: 80.0%). No response-related differences in hippocampal volume were detected, either at baseline or longitudinally.

**Figure 3:**
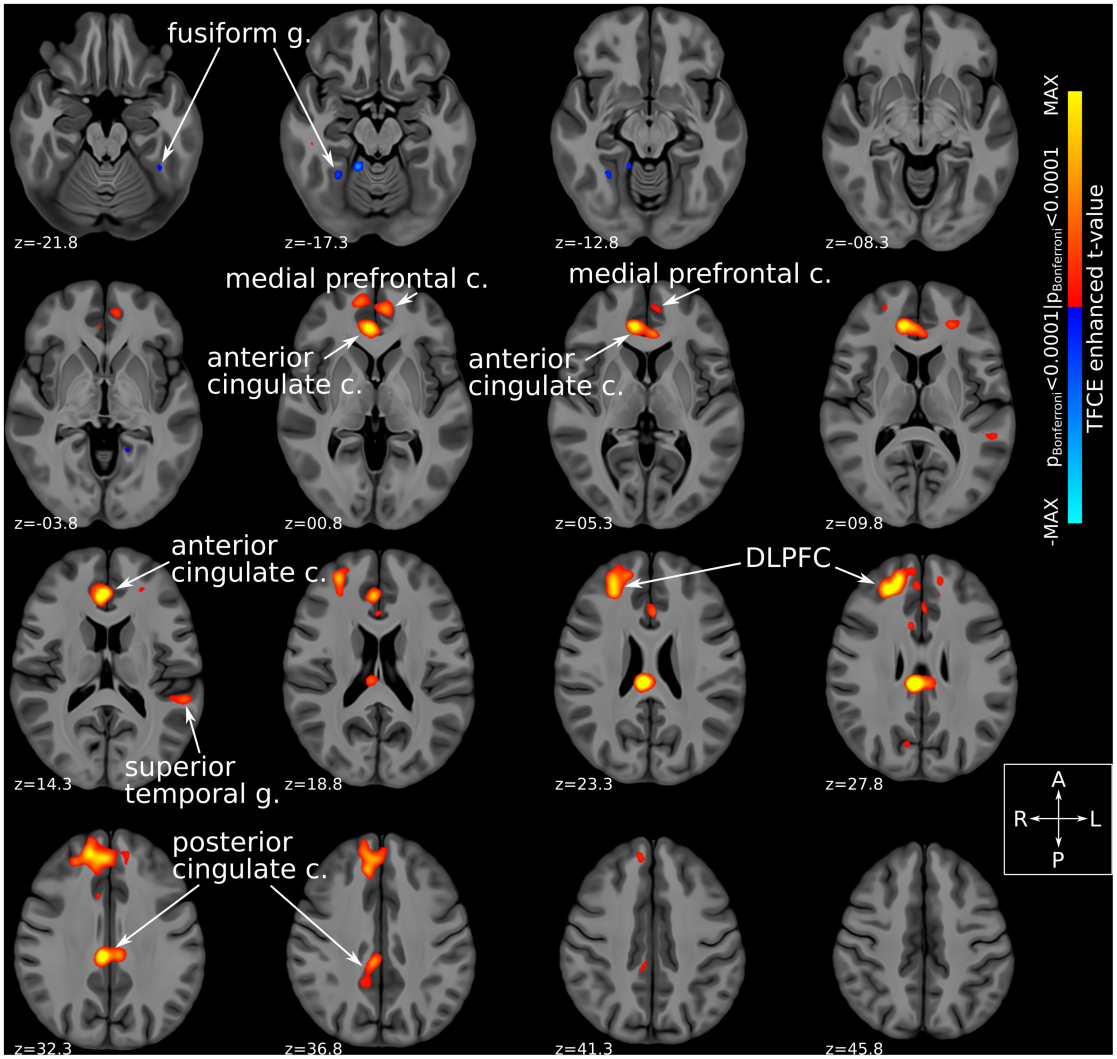
Habenular functional connectivity changes with active SCC-DBS. Change in habenular functional connectivity between DBS-ON and DBS-OFF states. Brain areas that showed a significant change in functional connectivity [TFCE-adjusted *p*_Bonferroni_ < 0.0001 (52)] between these states are superimposed on axial slices of a high resolution, high contrast brain template (53) in MNI standard space (42). Red/yellow colors denote areas that were more functionally connected with the bilateral habenula in the DBS-ON condition, while blue colors denote areas that showed less habenular connectivity in the DBS-ON state. Many of the areas whose habenular connectivity changed with SCC-DBS are known to be part of the “brain-wide network of depression.” *c*., cortex; *DBS*, deep brain stimulation; *DLPFC*, dorsolateral prefrontal cortex; *g*., gyrus; *MNI*, Montreal Neurological Institute; *SCC*, subcallosal cingulate area; *TFCE*, threshold-free cluster enhancement.

Comparison of fMRI-derived functional connectivity maps between DBS-ON and DBS-OFF states uncovered a number of brain regions whose connectedness to the Hb was significantly (TFCE-adjusted *p*_Bonferroni_ < 0.0001) altered by SCC-DBS stimulation. Specifically, active stimulation appeared to increase Hb functional connectivity with several prefrontal and corticolimbic regions, including rostral and dorsal anterior cingulate cortex (ACC), posterior cingulate cortex (PCC), medial prefrontal cortex, and dorsolateral prefrontal cortex (dlPFC). In addition, increased and decreased Hb connectivity was noted with superior temporal gyrus and fusiform gyrus, respectively (Figure 3). Due to the small number of patients included in the prospective fMRI analysis (*n* = 12), we were unable to conduct a comparison of habenular connectivity changes between responders and non-responders.

## Discussion

The present study employed multimodal MRI techniques to specifically investigate the involvement of the habenula (Hb) in clinical response to SCC-DBS. Evidence supporting such an involvement was observed in two ways: (i) longitudinal Hb volume change following DBS treatment differed in SCC-DBS responders compared to non-responders; (ii) active SCC-DBS stimulation acutely modulated Hb's functional connectivity to a number of regions that are implicated in brain-wide depression networks (54–57). Building on existing ECT, ketamine, and standard pharmacotherapy imaging work (23, 25), these results strengthen the notion that Hb plays an important role in subserving clinical response to a variety of antidepressant therapies. They also fit with preliminary evidence that DBS directly targeting Hb itself may be a useful therapy for depression (58, 59) and various other refractory neuropsychiatric disorders (60).

Our MRI-based volumetric analysis demonstrated that the trajectory of Hb volume change following SCC-DBS differed according to individual treatment response. Specifically, clinical responders (patients who experienced ≥ 50% symptom reduction) tended to exhibit increased habenular volume over time, while non-responders showed the opposite trend. Long-term volume alterations have been previously demonstrated in patients receiving DBS for various indications including Parkinson's and Alzheimer's disease (61, 62), suggesting that part of this intervention's therapeutic effect is mediated by neuroplastic changes. Indeed, a prior data-driven study—using a different method for volumetric analysis—in this same SCC-DBS patient population (55) identified other (mostly cortical) regions whose trajectories of volume change over time corresponded to patient outcome. The phenomenon of Hb volume changes in the context of psychiatric interventions is also supported by existing evidence. Work by Savitz et al. (23) indicates that antidepressant and/or mood stabilizing pharmacotherapy can also lead to Hb volume increases, suggesting that this may be a common marker of clinical response.

Using 3T fMRI, we additionally observed that acute SCC-DBS was associated with immediate changes in Hb functional connectivity. These changes occurred within minutes of switching between DBS-ON and DBS-OFF states, and might be mediated by direct projections between Hb and the cingulate cortex and medial prefrontal region, which have been demonstrated in rodent tracer studies (63). Active SCC stimulation generally increased Hb connectivity with a number of depression-implicated brain areas such as the anterior (specifically rostral and dorsal anterior cingulate cortex, lying outside of the direct stimulation target area) and posterior cingulate cortices, medial prefrontal cortex, dorsolateral prefrontal cortex, superior temporal gyrus, and fusiform gyrus. Many of these, including posterior cingulate cortex (64), superior and middle temporal gyrus (65–68), medial prefrontal cortex (69, 70), and fusiform gyrus (71), are heavily implicated in depression and mood regulation. The rostral and dorsal anterior cingulate activity in particular have been identified as key predictors of pharmacotherapy success (72, 73). Moreover, previous voxel-wise, data-driven fMRI analyses of an overlapping SCC-DBS cohort detected significant amplitude of low frequency fluctuations (ALFF; a measure of spontaneous neural activity) alterations in dorsal anterior cingulate as well as posterior cingulate when comparing DBS-OFF and DBS-ON conditions (47). Dorsolateral prefrontal cortex hypoactivity in depression is substantiated by the antidepressant effect of repetitive transcranial magnetic stimulation (rTMS) targeting this area (74–77). Interestingly, the degree of symptom improvement following dorsolateral prefrontal cortex rTMS has been linked to the functional connectivity between this region and SCC (78). Overall, our observation that therapeutic stimulation alters Hb connectivity with these mood-implicated areas tentatively positions Hb as a key player in the brain-wide network of depression (49–52). In particular, Hb may be important for mediating the expression of anhedonia across these circuits (79).

### Limitations

This study identified significant post-DBS changes in Hb volume that were not appreciated in a prior volumetric analysis of the same patient cohort (55). This apparent discrepancy may be explained by several factors, including different experimental designs and different methods used to discern longitudinal volume change. The former paper employed a data-driven, hypothesis-free approach (deformation-based morphometry) in which statistical tests were conducted at the voxel level, necessitating stringent multiple comparison correction. By contrast, the current study used the MAGeT segmentation algorithm to specifically estimate bilateral Hb volume in an *a priori* fashion. Finally, while our analyses are novel, they were conducted in relatively small-to-moderately sized patient cohorts (*n* = 32 for the volumetric analysis; *n* = 12 for the fMRI analysis). As such, the results outlined here are preliminary and should be confirmed in future work.

To conclude, this study lends support to growing evidence that Hb plays an important role in response to antidepressant therapies (26), suggesting that both structural and functional Hb features may contribute to neurobiological signatures of response to SCC-DBS. In doing so, it also strengthens the case for further exploration of DBS targeting Hb itself for refractory psychiatric disorders.

## Data Availability Statement

The raw data supporting the conclusions of this article will be made available by the authors, without undue reservation.

## Ethics Statement

All aspects of this study involving human patients were reviewed and approved by the University Health Network Research Ethics Board (REB). Patients participating in the prospective component of this study provided their written informed consent to do so.

## Author Contributions

GE and JG conceived and designed the study. GE, JG, MB, AP, BW, PG, VB, and SK contributed to data acquisition. GE, JG, AL, and AB contributed to data analysis. GE, JG, AL, and AB contributed to the initial draft of the manuscript and preparation of the figures. AML supervised the study. All authors reviewed and edited the manuscript.

## Funding

This study was supported by the RR Tasker Chair in Functional Neurosurgery at University Health Network (AML) and the Canadian Institutes of Health Research (reference # 164235: GE).

## Conflict of Interest

AML is the co-founder of Functional Neuromodulation (a DBS-related company), is a consultant for Boston Scientific, Medtronic, and Abbott (companies that produce DBS hardware). PG and SK are consultants for Abbott. GE, AB, and AML have intellectual property in the field of DBS. The remaining authors declare that the research was conducted in the absence of any commercial or financial relationships that could be construed as a potential conflict of interest.

## Publisher's Note

All claims expressed in this article are solely those of the authors and do not necessarily represent those of their affiliated organizations, or those of the publisher, the editors and the reviewers. Any product that may be evaluated in this article, or claim that may be made by its manufacturer, is not guaranteed or endorsed by the publisher.

## Supplementary Material

Supplementary Figure 1 Study flowchart. Three separate analyses were performed: a retrospective baseline volume analysis, a retrospective longitudinal volume analysis, and a prospective fMRI functional connectivity analysis. *DBS*, deep brain stimulation; *FC*, functional connectivity; *SCC*, subcallosal cingulate area.

Supplementary Figure 2 Habenular segmentation. **(A)** Segmented habenula illustrated on orthogonal slices of the MNI152 brain. This bilateral habenula label was used as a seed for the functional connectivity analysis. **(B)** Exemplar MAGeT habenula segmentation illustrated in orthogonal slices. *MNI*, Montreal Neurological Institute.

Supplementary Table 1 Imaging acquisition parameters for structural MR imaging.

Supplementary Table 2 Imaging acquisition parameters for functional MR imaging.
