# Supplementary figures and images for: Habenular Involvement in Response to Subcallosal Cingulate Deep Brain Stimulation for Depression
Source: Front Psychiatry. 2022 Feb 4;13:810777. doi: 10.3389/fpsyt.2022.810777 (PMC8854862; doi:10.3389/fpsyt.2022.810777)

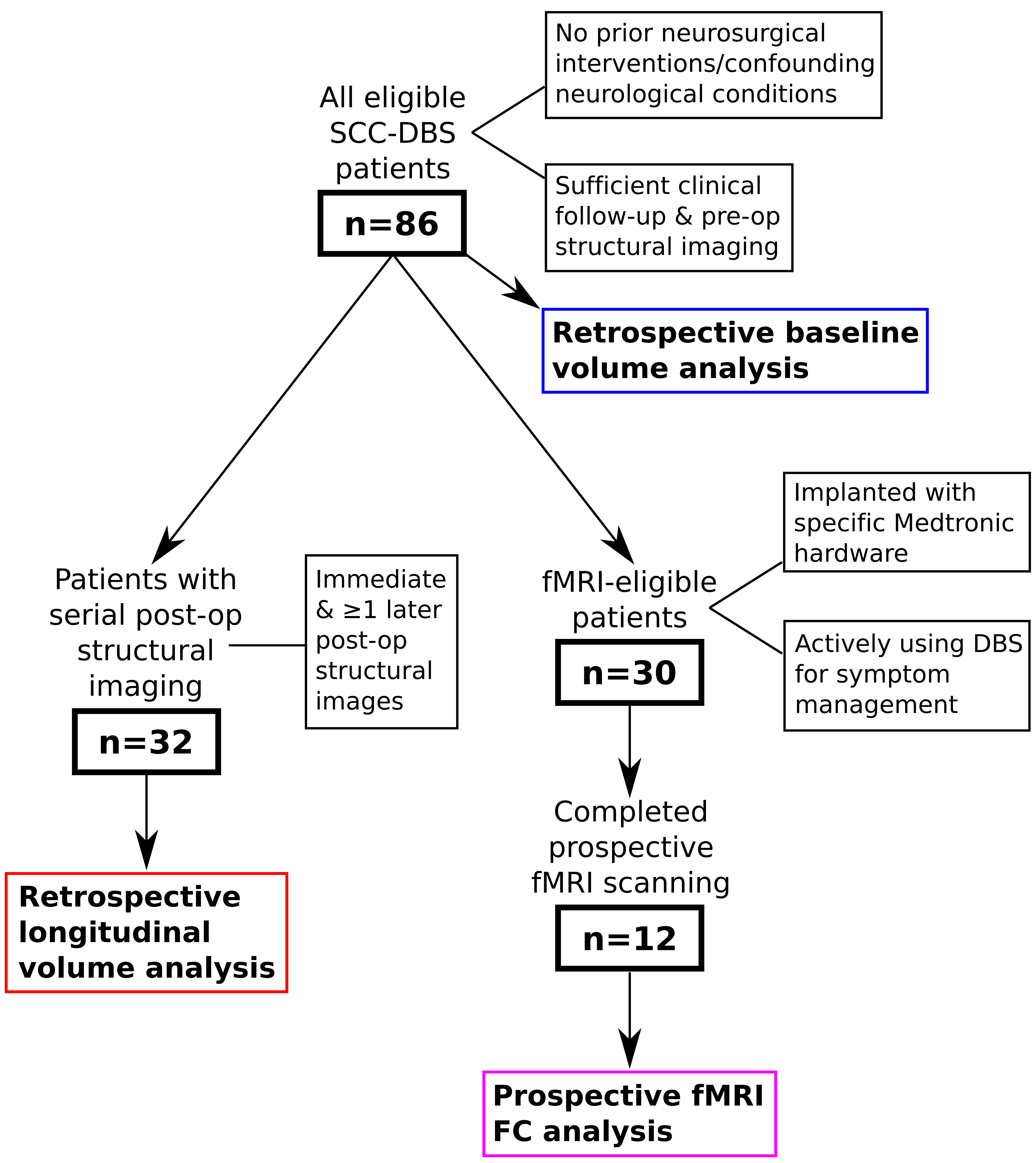

Supplement: Supplementary Figure 1 — Study flowchart. Three separate analyses were performed: a retrospective baseline volume analysis, a retrospective longitudinal volume analysis, and a prospective fMRI functional connectivity analysis. DBS, deep brain stimulation; FC, functional connectivity; SCC, subcallosal cingulate area. [file Image_1.TIF]

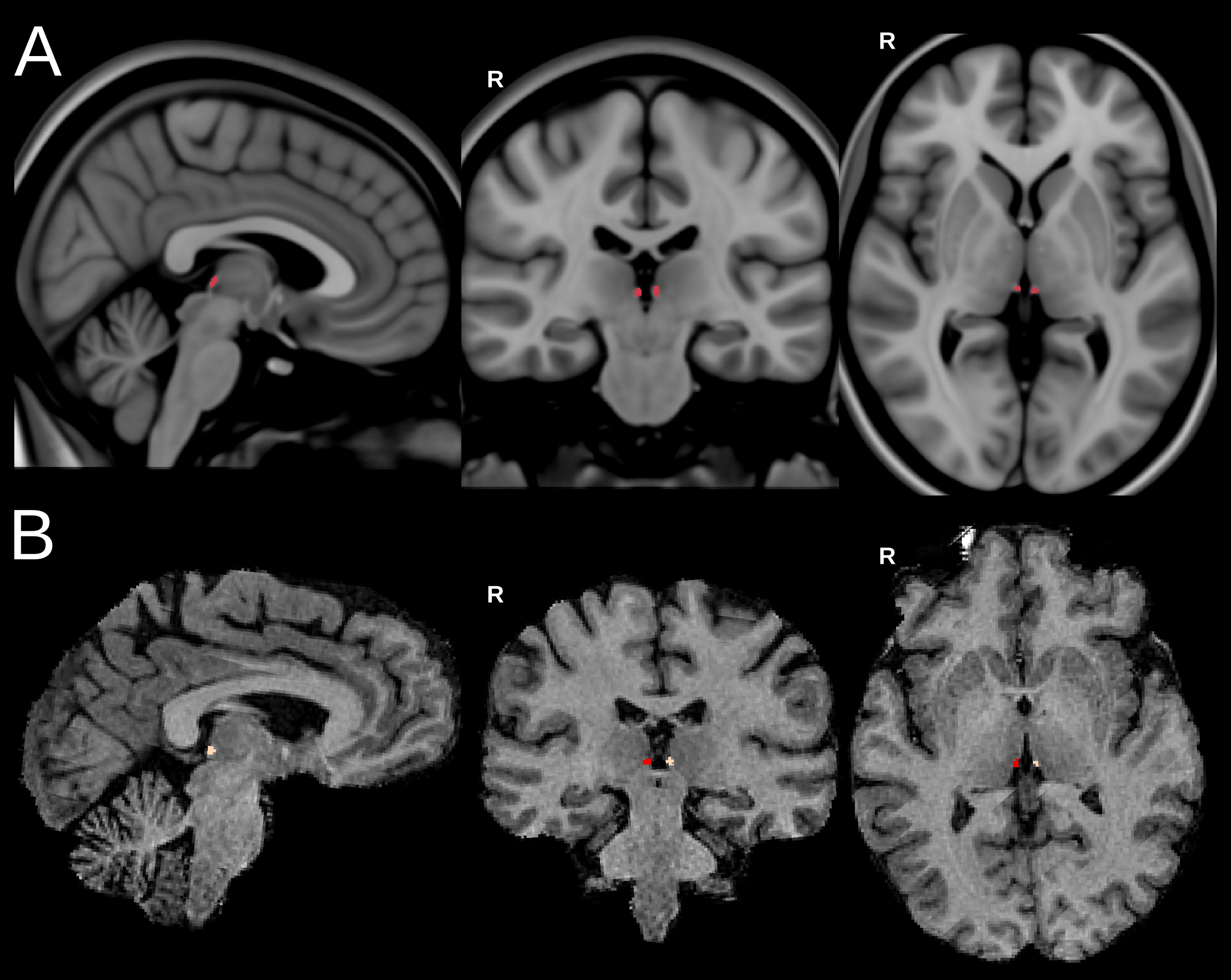

Supplement: Supplementary Figure 2 — Habenular segmentation. (A) Segmented habenula illustrated on orthogonal slices of the MNI152 brain. This bilateral habenula label was used as a seed for the functional connectivity analysis. (B) Exemplar MAGeT habenula segmentation illustrated in orthogonal slices. MNI, Montreal Neurological Institute. [file Image_2.TIFF]
