# Supplementary material for: Habenular Involvement in Response to Subcallosal Cingulate Deep Brain Stimulation for Depression
Source: Front Psychiatry. 2022 Feb 4;13:810777. doi: 10.3389/fpsyt.2022.810777 (PMC8854862; doi:10.3389/fpsyt.2022.810777)
Supplement: Supplementary Table 1 — Imaging acquisition parameters for structural MR imaging. [file Table_1.DOCX]

*Supplementary Table 1*. Imaging acquisition parameters for structural MR imaging.

| **Sequence** | **Scanner** | **Voxel size (mm)** | **TR (ms)** | **TE (ms)** | **Flip angle (°)** |
| --- | --- | --- | --- | --- | --- |
| *Pre-operative 3D-SPGR* | 1.5T GE Signa Excite | 1×1×1 | 12.4 | 5.3 | 20 |
| *Post-operative 3D-SPGR* | 1.5T GE Signa Excite | 1×1×1 | 11.9 | 5.0 | 20 |
|  | 3.0T GE Signa HDxt | 1×1×1 | 8.0 | 3.0 | 20 |

*GE*: General Electric; *SPGR*: spoiled gradient recalled; *1.5T*: 1.5 Tesla; *3.0T*: 3.0 Tesla.
