# Supplementary material for: Habenular Involvement in Response to Subcallosal Cingulate Deep Brain Stimulation for Depression
Source: Front Psychiatry. 2022 Feb 4;13:810777. doi: 10.3389/fpsyt.2022.810777 (PMC8854862; doi:10.3389/fpsyt.2022.810777)
Supplement: Supplementary Table 2 — Imaging acquisition parameters for functional MR imaging. [file Table_2.DOCX]

*Supplementary Table 2.* Imaging acquisition parameters for functional MR imaging.

| **Sequence (coil)** | **TR (ms)** | **TE (ms)** | **TI (ms)** | **BW (kHz)** | **FOV (mm)** | **FA (°)** | **ST (mm)** | **Gap (mm)** | **ETL** | **Matrix (voxels)** | **Frequency direction** | **NEX** |
| --- | --- | --- | --- | --- | --- | --- | --- | --- | --- | --- | --- | --- |
| *GRE-EPI multiphase fMRI (body coil)* | 2151 | 30 | N/A | 62.5 | 240×240 | 76 | 4 | 0 | 1 | 64×64 | L/R | 1 |
| *GRE-EPI multiphase fMRI (T/R coil)* | 3010 | 30 | N/A | 62.5 | 240×240 | 84 | 3 | 0 | 1 | 64×64 | L/R | 1 |
| *3D-SPGR T1w (T/R coil or body coil)* | 8 | 3 | 450 | 31.25 | 256×256 | 20 | 1 | 0 | 1 | 256×256 | A/P | 1 |
|  |  |  |  |  |  |  |  |  |  |  |  |  |

All sequences were acquired using a 3T GE Signa HDxt scanner. Structural T1w sequences and functional MR sequences were acquired using a body-transmit coil (GE model 2380637-2) in 11 patients and a quadrature birdcage transmit-receive (T/R) coil (GE model 2376114) in one patient. *A/P*: anterior-to-posterior; *BW*: bandwidth; *GRE-EPI*: gradient-recalled echo echo-planar imaging; *ETL*: echo train length; *FA*: flip angle; *FOV*: field of view; *GE*: General Electric; *kHz*: kilohertz; *L/R*: left-to-right; *N/A*: not available; *NEX*: number of excitations; *SPGR*: spoiled gradient-recalled; *ST*: slice thickness; *TE*: echo time; *TI*: inversion time; *TR*: repetition time; *T1w*: T1-weighted; *3T*: 3.0 Tesla.
